# Gender differences in clinical features, comorbidities and prognostic outcomes in idiopathic pulmonary fibrosis—a retrospective cohort analysis from the British Thoracic Society Interstitial Lung Disease Registry

**DOI:** 10.1136/bmjopen-2025-104914

**Published:** 2025-10-28

**Authors:** Leng Cheng Sia, Gina Amanda, Karol Bączek, Andrew Achaiah, Lucile Sesé, Nazia Chaudhuri, On Behalf of the British Thoracic Society Interstitial Lung Disease Registry Steering Group, Graham Bloye

**Affiliations:** 1 Respiratory Medicine, University Malaya Medical Centre, Federal Territory of Kuala Lumpur, Malaysia; 2 Universiti Malaya, Federal Territory of Kuala Lumpur, Malaysia; 3 Department of Medicine, Jakarta Islamic Hospital Cempaka Putih, Jakarta, Indonesia; 4 Department of Pneumology, Medical University of Lodz, Łódź, Poland; 5 Gloucestershire Hospitals NHS Foundation Trust, Cheltenham, UK; 6 Hôpital Avicenne, Bobigny, France; 7 Department of Life and Health Sciences, Ulster University, Coleraine, UK

**Keywords:** Fibrosis, REGISTRIES, Interstitial lung disease

## Abstract

**Abstract:**

**Background:**

Idiopathic pulmonary fibrosis (IPF), an unknown aetiology type of interstitial lung disease (ILD), carries the poorest prognosis and is more common in males and the elderly. Gender differences in baseline presentation, lung function and comorbidities may have an impact on prognostic outcomes.

**Objective:**

The aim of this study was to explore gender differences in clinical features, comorbidities and outcomes in IPF in a UK cohort.

**Method:**

This was a retrospective cohort study analysing data from the British Thoracic Society UK IPF ILD Registry from January 2013 to February 2024. We compared baseline characteristics between males and females, and a survival analysis in both genders was performed using the Cox proportional hazards model.

**Results:**

We identified 6666 IPF patients with a mean age at diagnosis of 74.1±8.1. Our cohort was predominantly male (5197, 78%), with a higher proportion of current and ex-smokers compared with females (69.9% vs 59.9%, p<0.001) and higher rates of comorbidities such as ischaemic heart disease (IHD) and diabetes (19.7% vs 14.6% and 19.9% vs 11.2%, respectively, p<0.001). Baseline forced vital capacity (FVC) % predicted was 77.76±17.4 in males and 81.83±19.7 in females (p=0.001), while diffusing capacity for carbon monoxide (DLCO) was similar between the two groups. In multivariate analysis, after adjusting for age, IHD and lower baseline FVC, DLCO was a poor survival predictor in males. Hiatus hernia is a protective factor. Conversely, disease duration of <12 months, gastro-oesophageal reflux disease, not requiring oxygen at baseline and higher baseline DLCO predicted better survival in females.

**Conclusion:**

Gender differences in baseline characteristics and prognostic factors were observed in IPF. A gender-based approach in managing IPF is warranted, and further studies are needed to clarify these differences and their impact on IPF management.

**STRENGTHS AND LIMITATIONS OF THIS STUDY**

- A large cohort with over 6666 patients with idiopathic pulmonary fibrosis in the UK, specifically aimed at identifying gender differences in presentations and outcomes.
- Approximately 22% of female patients (n=1470) were included, which allowed a meaningful comparison.
- This was a retrospective study using real-world data, meaning missing data and observer bias are potential limitations.

## Introduction

 Idiopathic pulmonary fibrosis (IPF) is an unknown type of interstitial lung disease (ILD) characterised by chronic progressive and irreversible lung scarring.^1^,^3^ Globally, the adjusted incidence and prevalence of IPF per 10 000 population are in the range of 0.09–1.3 and 0.33–4.51, respectively.^1^ Patients with IPF also have poor prognosis, with 3-year and 5-year survival rates of 61% and 45%, respectively.^2^ The pathogenesis of IPF is complex, with an interplay between genetics and the environment. Several factors have been attributed to the risk of developing IPF, including advanced age, genetic factors such as short telomere genes or MUC5B polymorphism, male gender and environmental exposures such as smoking, pollution and the lung microbiome. Comorbidities such as gastro-oesophageal reflux disease (GERD), obstructive sleep apnoea, diabetes mellitus and chronic viral infection have also been highlighted as contributing to the natural history of IPF.^4^

Several studies demonstrate that IPF is predominant in males, approximating 67%–78%.^3^,^6^ The potential cause of this male predominance is poorly understood. Studies have reported reduced testosterone levels and increased oestrogen receptor alpha in males with IPF.^7^,^9^ In addition, lower plasma testosterone levels correlate with short leucocyte telomere length in male IPF patients.^8^ These findings may suggest a role for sex hormones in gender differences in IPF. Furthermore, differences in environmental exposures that predominate between genders may also interact and contribute to this risk. Males have a higher smoking frequency and occupational exposures than females, and this may contribute to their increased incidence of IPF.^4 5 10^

Growing evidence of gender differences in IPF has led to several national studies exploring this association, with mixed findings. A study from France reported lower baseline forced vital capacity (FVC) in females, whereas a Swedish study observed higher FVC in females.^3 11^ Some cohort studies from the US) and Italy found that males presented at an older age compared with females.^12 13^ Radiological differences have been investigated in a French registry.^11^ However, evidence regarding gender differences in comorbidities and their impact on survival remains limited.

Mortality rates in IPF are consistently higher in males than in females across multiple national cohorts.^1214^,^16^ Males with IPF also have worse transplant-free survival than females, with approximately a difference of 13 months in median transplant-free survival between genders.^12^

The reasons underlying these differences are likely multifactorial, including genetic, hormonal, behavioural, environmental and socioeconomic influences. Potential inequities in presentation, diagnosis and treatment access may further contribute to gender disparities in outcomes. Evidence regarding gender differences in comorbidities and their impact on survival remains limited. We hypothesised that gender differences would be evident in baseline characteristics, comorbidities and prognostic outcomes in a UK IPF population. By addressing this knowledge gap, our study aims to inform the development of more equitable, gender-sensitive management strategies.

## Methodology

### Study design and data source

This was a retrospective review analysing data from a prospectively maintained British Thoracic Society (BTS) UK IPF ILD Registry. This national registry, established in 2013, includes participation from 91 hospital sites across 74 health boards across the UK, with 26 English prescribing centres commissioned by NHS England as specialist ILD centres.^17^ Written informed consent was obtained from all participants. Data were entered via a purpose-designed web platform (https://registry.brit-thoracic.org.uk/). Data access was granted through data-sharing agreements, and ethical approval was obtained from the National Research Ethics Service (Reference: 22/EE/0235).

### Study population

The BTS UK IPF ILD registry includes patients with a multi-disciplinary diagnosis of IPF. Inclusion criteria include patients first seen at participating sites from January 2013 with a new or historic diagnosis of definite or strongly suspected IPF based on clinical characteristics with unknown cause. Patient data included patients from 1 January 2013 to the censor date of 1 February 2024.

### Patient and public involvement

Patients and the public were involved in developing the BTS Lung Disease Registry Programme through Asthma+Lung UK, Sarcoidosis UK, and a lay member of the BTS Board of Trustees. Their views on governance, design and communication remain part of the programme, with ongoing input from Action for Pulmonary Fibrosis, Sarcoidosis UK and oversight by the BTS Board.

### Baseline data collection

Data collection encompassed baseline demographic and clinical information including age at diagnosis, gender, duration of chest symptoms prior to baseline visit and comorbidities, including diabetes, hypertension, ischaemic heart disease (IHD), arrhythmia, valvular heart disease, left ventricular failure, lung cancer, chronic obstructive pulmonary disease, malignancy, major depressive disorder, GERD, hiatus hernia (HH) and without comorbidities. Lung function parameters, specifically FVC and diffusing capacity for carbon monoxide (DLCO), as well as radiological patterns, lung biopsy results and treatment history, were also recorded. The Index of Multiple Deprivation (IMD) was provided. The IMD is based on seven deprivation domains: income, employment, education, health, crime, housing and services and living environment, reflecting the socioeconomic status of the population.^18^ IMD was divided into 5 subgroups from 1 to 5, with one being the most deprived and five being the least deprived.

Comorbidities with a prevalence of <2% were excluded from the analysis (online supplemental table S1).^19^ Chest symptoms prior to baseline visit were sub-grouped into two categories: <12 months and more than 12 months. Radiological patterns were reclassified as definite usual interstitial pneumonia (UIP), characterised by the presence of honeycombing, probable UIP, and a combined category of indeterminate UIP and alternative diagnosis.^20^

### Outcome measures

The differences between males and females in baseline characteristics, duration of chest symptoms, lung function tests, radiological patterns, lung biopsy, treatment and mortality outcome were compared. Overall survival was defined in years from the date of baseline visit to the date of death from any cause. Records with disease durations exceeding 100 years, DLCO values >150% or <1% of the predicted value, FVC values >200% of the predicted value, or ages >150 or <10 years at diagnosis were considered outliers or errors in data entry and treated as missing data. This approach was employed to ensure data accuracy and integrity by excluding extreme or biologically implausible values from the analysis.

### Data analysis and statistics

Statistical analyses were performed using IBM SPSS Statistics (V.21.0). Missing values were handled pairwise without imputation. Continuous variables were reported as mean±SD while categorical variables were presented as numbers and percentages. Normality of continuous variables was assessed based on skewness and kurtosis, with thresholds of ±2 and ±3, respectively, indicating acceptable normality. Gender (Males vs females) was compared using χ² tests for categorical variables, independent t-tests for normally distributed continuous variables and Mann-Whitney U tests for continuous variables not normally distributed. A p value of <0.05 was considered statistically significant. The median survivals between men and women were compared by using a log-rank test. To identify factors associated with survival in men and women, Cox regression models were performed for each gender. Variables from univariate analyses with p<0.05 were recruited for multivariate Cox-regression hazard analysis and adjusted for age.

## Result

### Sample population

Among 6838 IPF cases, 6666 patients were included after excluding those with missing gender data. A comparison of baseline characteristics, comorbidities, IMD, lung function tests, radiological patterns and treatments among both genders is shown in table 1. Males comprised 5196 (78%), with a mean age of 74.1±8.1; 4311 (67.8%) were current or ex-smokers, and 3918 (63.7%) presented with more than 12 months of chest symptoms prior to baseline visit. In terms of socioeconomic status, 1025 participants (16.8%) were categorised as most deprived (IMD 1), and 1473 participants (24.1%) were categorised as least deprived (IMD 5). While 34.7% had no comorbidities, the most common comorbidities were hypertension (33.4%), diabetes (18.6%), IHD (18%), GERD (17.4%) and HH (6.8%). Arrhythmia was present in 4.9% of the patients. The mean FVC was 78.7±18.0, whereas the mean DLCO was 50.7±16.8. A total of 2400 (42.6%) patients had a definite UIP pattern on high-resolution CT (HRCT). Furthermore, 6035 (90.5%) patients had a multidisciplinary team diagnosis of IPF. On baseline visit, 3033 (45.7%) had been on or were started on antifibrotic drugs, while 1108 (16.6%) had been or were started on oxygen therapy.

**Table 1:** Baseline data comparing males and females

Characteristic	Overall	Male	Female	P value
Age at presentation	74.1±8.1	74.1±8.1	73.9±8.1	0.557
Smoking status				<0.001*
Yes	4311 (67.8)	3482 (69.9)	829 (59.9)	
Current	216 (3.4)	176 (3.5)	40 (2.9)	
Ever smoker	4095 (64.4)	3306 (66.4)	789 (57.0)	
Never	2052 (32.2)	1498 (30.1)	554 (40.1)	
Comorbidities				
None	2312 (34.7)	1740 (33.5)	572 (38.9)	<0.001*
Diabetes	1237 (18.6)	1023 (19.7)	214 (14.6)	<0.001*
Hypertension	2225 (33.4)	1753 (33.7)	472 (32.1)	0.242
IHD	1200 (18)	1035 (19.9)	165 (11.2)	<0.001*
Arrhythmia	328 (4.9)	270 (5.2)	58 (3.9)	0.050
GERD	1157 (17.4)	884 (17.0)	273 (18.6)	0.164
Hiatus hernia	450 (6.8)	332 (6.4)	118 (8.0)	0.027*
Lung Function				
FVC	78.7±18.0	77.76±17.4	81.83±19.7	<0.001*
DLCO	50.7±16.8	50.64±16.9	50.65±16.4	0.962
Duration of chest symptoms prior to baseline visit				0.147
<12 months	2234 (36.3)	1765 (36.8)	469 (34.6)	
More than 12 months	3918 (63.7)	3033 (63.2)	885 (65.4)	
IMD				0.006*
1 (Most deprived)	1025 (16.8)	793 (16.6)	232 (17.2)	
2	1000 (16.4)	740 (15.5)	260 (19.3)	
3	1239 (20.3)	964 (20.2)	275 (20.4)	
4	1376 (22.5)	1097 (23.0)	279 (20.7)	
5 (Least deprived)	1473 (24.1)	1174 (24.6)	299 (22.2)	
HRCT pattern				0.301
Definite UIP	2400 (42.6)	1887 (42.8)	513 (41.9)	
Probable UIP	2939 (52.2)	2280 (51.7)	659 (53.8)	
Indeterminate and alternative diagnosis	296 (5.3)	243 (5.5)	53 (4.3)	
Lung biopsy	28 (0.4)	22 (0.4)	6 (0.4)	0.926
MDT discussion				0.044*
Yes	6035 (90.5)	4727 (91.0)	1308 (89.0)	
No	307 (4.6)	222 (4.3)	85 (5.8)	
Awaiting	153 (2.3)	121 (2.3)	32 (2.2)	
Not known	171 (2.6)	126 (2.4)	45 (3.1)	
Treatment				
Antifibrotic	3050 (46)	2444 (47.2)	610 (41.6)	<0.001*
Pirfenidone	1323 (19.9)	1082 (20.9)	261 (17.9)	
Nintedanib	1710 (25.8)	1377 (26.6)	354 (24.2)	
History of both	21 (0.3)	15 (0.3)	6 (0.4)	
Immunosuppressant	335 (5.2)	248 (4.9)	87 (6.1)	0.071
Baseline oxygen requirement	1108 (16.6)	851 (16.4)	257 (17.5)	0.315

Data are presented as n (%) or mean±SD unless otherwise stated.

* p<0.05.

DLCO, diffusion of lung capacity for carbon monoxide; FVC, forced vital capacity; GERD, gastro-oesophageal reflux disease; HRCT, high-resolution CT; IHD, ischaemic heart disease; IMD, index of multiple deprivation; MDT, multi-disciplinary team discussion; UIP, usual interstitial pneumonia.

### Baseline characteristics comparing males and females

The mean ages for males and females at baseline visit were similar, 74.1±8.1 and 73.9±8.1, respectively (p=0.557). Current and ex-smokers were significantly more prevalent in males compared with females (69.9% vs 59.9%, p<0.001). Diabetes and IHD were more common in males (19.7% vs 14.6% and 19.9% vs 11.2%, respectively, p<0.001). Conversely, HH was more prevalent in females (6.4% vs 8.0%, p=0.027), and more females had no comorbidities (33.5% vs 38.9%, p<0.001). Males were less deprived, with a higher percentage in the IMD 4–5 category compared with females (43.7% vs 39.3%, p<0.006). Both genders had similar durations of chest symptoms prior to the clinic visit. Males had a lower baseline FVC than females (77.76±17.4 vs 81.83±19.7, p=0.001); however, no significant difference was observed in DLCO. There was no difference in baseline radiological patterns. A higher proportion of males was on antifibrotic therapy compared with females (47.2% vs 41.6%, p<0.001).

### Univariate and multivariate analysis for outcomes between males and females

Table 2 demonstrates univariate Cox regression analysis performed separately for males and females to identify factors associated with survival. Significant variables from this analysis were selected for inclusion in the multivariate model.

**Table 2:** Univariate Cox regression analysis among males and females

Baseline data	Male n=5196				Female n=1470			
	Number	HR	95% CI	P value	Number	HR	95% CI	P value
Age at presentation	5144	1.02	1.01 to 1.02	<0.001*	1435	1.01	1.00 to 1.03	0.083
Smoking	4940	1.19	1.06 to 1.33	0.004*	1345	1.12	0.90 to 1.39	0.306
Comorbidities								
None	5152	1.03	0.92 to 1.14	0.639	1437	0.87	0.70 to 1.07	0.192
Diabetes	5152	1.11	0.98 to 1.25	0.109	1437	1.08	0.82 to 1.43	0.575
Hypertension	5152	0.79	0.89 to 1.10	0.788	1437	1.05	0.84 to 1.30	0.687
IHD	5152	1.29	1.15 to 1.44	<0.001*	1437	1.11	0.82 to 1.50	0.509
Arrhythmia	5152	0.85	0.64 to 1.11	0.227	1437	1.03	0.56 to 1.87	0.934
GERD	5152	0.83	0.73 to 0.95	0.008*	1437	0.65	0.49 to 0.87	0.003*
Hiatus hernia	5152	0.68	0.54 to 0.86	0.001*	1437	0.64	0.41 to 1.00	0.054
Baseline oxygen requirement	5152	1.76	1.56 to 1.98	<0.001*	1437	1.86	1.47 to 2.36	<0.001*
More than 12 months chest symptoms prior to baseline visit	4761	1.08	0.97 to 1.21	0.147	1328	1.43	1.13 to 1.81	0.003*
HRCT	4377			<0.001*	1201			0.040*
Definite UIP	1877	1	1	–		1	1	–
Probable UIP	2257	0.78	0.70 to 0.87	<0.001		0.75	0.60 to 0.94	0.012
Indeterminate and alternative	243	0.76	0.60 to 0.96	0.022		0.96	0.59 to 1.57	0.883
Lung function								
FVC	4913	0.98	0.98 to 0.99	<0.001*	1372	0.99	0.99 to 1.00	0.006*
DLCO	3529	0.97	0.97 to 0.98	<0.001*	891	0.97	0.97 to 0.98	<0.001*
IMD	4727			0.003*	1320			0.038*
1	789	1	1		230	1	1	
2	736	0.97	0.82 to 1.15	0.718	258	1.17	0.84 to 1.63	0.355
3	953	0.75	0.63 to 0.88	<0.001*	272	0.82	0.57 to 1.17	0.277
4	1090	0.86	0.73 to 1.00	0.056	276	0.89	0.63 to 1.26	0.511
5	1169	0.81	0.69 to 0.95	0.009*	294	0.70	0.490 to 1.005	0.053

* p<0.05.

DLCO, diffusion of lung capacity for carbon monoxide; FVC, forced vital capacity; GERD, gastro-oesophageal reflux disease; HRCT, high-resolution CT; IHD, ischaemic heart disease; IMD, index of multiple deprivation; UIP, usual interstitial pneumonia.

Table 3 shows the Cox regression analysis for both genders. In males, increasing age at presentation (HR 1.02, 95% CI 1.01 to 1.03, p<0.001), presence of IHD (HR 1.21, 95% CI 1.05 to 1.40, p=0.009), and lower FVC (HR 0.99, 95% CI 0.98 to 0.99, p<0.001) and DLCO (HR 0.98, 95% CI 0.97 to 0.98, p<0.001) were significantly associated with poorer survival. HH was a protective factor in males (HR 0.74, 95% CI 0.56 to 0.97, p=0.031). In females, duration >12 months (HR 1.61, 95% CI 1.18 to 2.20, p=0.003), oxygen requirement at baseline (HR 1.46, 95% CI 1.02 to 2.04, p=0.041), and lower DLCO (HR 0.98, 95% CI 0.97 to 0.99, p<0.001) were significantly associated with poor survival. GERD (HR 0.58, 95% CI 0.40 to 0.84, p=0.005) was identified as a protective factor. IMD and radiological patterns showed no significant survival association in either sex. Males had a median survival of 8.3 years (95% CI 7.41 to 9.19), compared with 11.6 years for females. However, no CI could be calculated for females due to high rates of censoring. Survival differences between groups were statistically significant (log-rank χ²=17.486, p<0.001).

**Table 3:** Multivariate Cox regression analysis among males and females

Baseline data	Males			Females		
	HR	95% CI	P value	HR	95% CI	P value
Age at presentation	1.02	1.01 to 1.03	<0.001*	1.03	1.01 to 1.05	0.001*
Smoking	1.12	0.97 to 1.31	0.123	–	–	–
More than 12 months chest symptoms prior to clinic	–	–	–	1.61	1.18 to 2.20	0.003*
IHD	1.21	1.05 to 1.40	0.009*	–	–	–
GERD	0.89	0.75 to 1.05	0.166	0.58	0.40 to 0.84	0.004*
Hiatus hernia	0.74	0.56 to 0.97	0.031*	–	–	–
On oxygen therapy	1.05	0.89 to 1.24	0.592	1.45	1.02 to 2.04	0.036*
HRCT			0.377			0.232
Definite UIP	1	1		1	1	
Probable UIP	0.92	0.81 to 1.05	0.226	0.81	0.61 to 1.09	0.160
Indeterminate and alternative	1.05	0.80 to 1.37	0.724	1.23	0.64 to 2.38	0.539
FVC	0.99	0.98 to 0.99	<0.001*	1.00	0.99 to 1.01	0.659
DLCO	0.98	0.97 to 0.98	<0.001*	0.98	0.97 to 0.99	<0.001*
IMD			0.361			0.204
1	1	1	–	1	1	–
2	0.97	0.79 to 1.21	0.813	1.22	0.79 to 1.89	0.372
3	0.82	0.66 to 1.01	0.057	0.87	0.55 to 1.37	0.544
4	0.94	0.77 to 1.15	0.559	0.85	0.52 to 1.38	0.509
5	0.91	0.75 to 1.11	0.347	0.75	0.47 to 1.19	0.222

* p<0.05.

DLCO, diffusion of lung capacity for carbon monoxide; FVC, forced vital capacity; GERD, gastro-oesophageal reflux disease; IHD, ischaemic heart disease; IMD, index of multiple deprivation; MDT, multi-disciplinary team discussion; UIP, usual interstitial pneumonia.

## Discussion

In the BTS registry, during the baseline visit, males were less deprived, had a higher rate of smoking history and more comorbidities compared with females with IPF. Males also had lower FVC at presentation and were more frequently treated with antifibrotic medication than females. Prognostic determinants differed between males and females. After adjusting for age, males with IHD, lower baseline FVC and DLCO had poor survival, while the presence of HH appeared to be a protective factor. In contrast, the duration of chest symptoms >12 months, GERD, baseline oxygen requirement and low DLCO were associated with a poorer prognosis in females. These findings highlight distinct prognostic factors between males and females, suggesting the need for gender-specific approaches in the management of IPF.

We believe this is an analysis of one of the largest registries of IPF patients. We analysed over 6666 patients with IPF in the UK with the specific purpose of identifying any gender differences in presentations and outcomes. IPF is a male-predominant disease, and this is representative in our data with 78% male representation, similar to all the national registries.^36 21^,^24^ Despite this, 1470 female patients with IPF were enrolled into the BTS IPF registry, which provides meaningful comparison.

The higher prevalence of smoking, IHD and diabetes among males with IPF in our study was comparable to several studies.^3 12^ This finding is not surprising, given the higher proportion of male smokers in the UK population.^25^ Additionally, IHD and diabetes are more commonly observed in males in the general population.^26 27^ The higher prevalence of HH among females is seen in some general population studies, though findings are inconsistent. In IPF, data are limited and based on CT-detected cases rather than true population prevalence.^28^ No significant difference was found in terms of age at diagnosis between the two genders, which is consistent with findings from other studies.^5 29^ This is expected as the duration of disease presentation did not differ significantly between males and females, suggesting the onset of disease is likely across genders.

Males exhibited lower baseline FVC compared with females, and lower baseline FVC and DLCO represented poor survival in this group. The prognostic value of baseline FVC aligns with findings from French and Swedish registries, as well as a cohort study focused on gender differences in IPF.^3 5 12^ Conversely, no gender differences in DLCO were observed. Yet, baseline DLCO, but not FVC, was significantly associated with survival in females. This is similar to a study by Mogulkoc *et al*, which reported DLCO- but not FVC- as a significant predictor of survival in IPF overall.^30^ The registry data from tertiary centres in the US show similar findings in males but not females.^12^ While longitudinal changes in FVC provide a more reliable predictor of mortality, baseline lung physiology such as FVC and DLCO remains important indicators of mortality, alongside age and gender.^31^,^33^ Cottin *et al* proposed that the relative preserved FVC in females may be due to a higher combined pulmonary fibrosis and emphysema index in females, indicating more extensive emphysema, although emphysema data were not available in our cohort as well as in the US cohort.^11 12^ DLCO may thus be a more reliable baseline lung physiology predictor in females, although it can also be influenced by other pathologies such as pulmonary vascular disease or pulmonary oedema.^34 35^

In our cohort, males were more frequently treated with antifibrotic treatment than females. FVC was lower in males, with values typically <78% but >80% in females. Similarly, studies from the US also report that fewer females receive antifibrotic treatment.^36 37^ Despite similar duration of symptoms and HRCT findings between genders, indicating no diagnostic delay or imaging-based bias, prescription patterns differed. Health-seeking behaviour is therefore unlikely to explain the disparity. Instead, antifibrotic prescribing in the UK was historically restricted to patients with an FVC <80% between 2013 and 2023, under the National Institute for Health and Care Excellence guideline. Nintedanib for patients with FVC >80% was initially deemed not cost-effective, as the incremental cost-effectiveness ratios exceeded the thresholds considered acceptable for National Health Service resources. Given that our cohort includes patients enrolled since 2013, these historical prescribing restrictions likely influenced treatment patterns and may have contributed to the observed gender differences.^38 39^

The survival paradox was evident in our cohort, as males had a median survival 3.3 years shorter than females despite being more frequently prescribed antifibrotic therapy. This real-world finding highlights that higher prescription rates do not necessarily translate into improved survival outcomes. Disease progression in IPF is not solely determined by antifibrotic use; other confounding factors play a crucial role. In our cohort, males presented with a higher comorbidity burden, lower baseline FVC and a greater history of smoking, all of which are associated with poorer prognosis.

Approximately two-thirds of patients presented with symptoms for more than 12 months prior to the baseline visit, with no significant differences between sexes. Among females, longer symptom duration was associated with a mean survival that was 3 years shorter compared with those who presented earlier (online supplemental table S3). Prolonged symptom duration may reflect delayed initiation of antifibrotic therapy. Hoyer *et al* reported that in patients with baseline FVC >80% predicted, longer diagnostic delay has a greater impact on progression-free survival after diagnosis.^40^ Our study supports this observation, as symptom duration appeared to have a stronger impact among females with higher baseline FVC in our cohort.

In contrast to a few studies reporting a high prevalence of HH, up to 42%–53% in IPF patients, our cohort demonstrated a significantly lower incidence of HH at 6.8%, with a higher prevalence observed in females.^28 41 42^ HH was captured prospectively by patient self-reported clinical diagnosis and clinician entry in a standardised proforma used across all centres since 2013, whereas published prevalence rates are typically derived from CT. As such, under-reporting is possible in our cohort. Tossier *et al* and Mackintosh *et al* highlight an association between HH and poor survival in IPF.^28 42^ However, our data suggest that the presence of HH in males may be a protective factor. Some studies found no clear link between HH and mortality, which might indicate that HH does not significantly impact survival outcomes in certain IPF cohorts.^41 43^ HH is a condition caused by translocation of the stomach above the diaphragm as well as widening of the oesophageal hiatus, which could be partly caused by degeneration over time affecting the compositional elastin and collagen fibres or due to pressure gradient in between intrathoracic and intra-abdominal.^44^ Interestingly, the presence of HH was significantly associated with higher baseline FVC in our cohort (online supplemental table S2). These conflicting data require further exploration.

GERD, closely related to HH, has been shown to be associated with improved survival.^41 45 46^ This is consistent with our findings, where GERD appeared to be associated with improved survival in females. Whether proton pump inhibitor treatment contributes to this effect remains uncertain and has been a topic of controversy for decades.

The present study poses a few limitations. First, this was a retrospective real-world data analysis of a national registry, which may pose potential observer bias. Second, data quality and consistency varied with the occurrence of incomplete or missing data, which may impact analysis and conclusions. This was mitigated somewhat by a standardised data set that was collected; however, it is evident that some questions could be left blank, which contributed to missing data and may bias the interpretation of results. Lastly, there is a lack of robustness of survival data as participating centres may not be informed of patients’ deaths and may not update the registry records.

## Conclusion

In conclusion, our findings emphasise the importance of a gender-specific approach to the diagnosis and management of IPF in view of distinct prognostic indicators between males and females. For example, earlier screening and referral pathways may be warranted in women presenting with unexplained dyspnoea or cough, whereas a male-specific focus could prioritise smoking cessation programmes and optimisation of cardiovascular risk. Future prospective studies are warranted to further explore these differences and evaluate their implications for IPF management.

## Supplementary material

10.1136/bmjopen-2025-104914 online supplemental table 1

## Data Availability

Data may be obtained from a third party and are not publicly available.
