# Supplementary material for: Gender differences in clinical features, comorbidities and prognostic outcomes in idiopathic pulmonary fibrosis—a retrospective cohort analysis from the British Thoracic Society Interstitial Lung Disease Registry
Source: BMJ Open. 2025 Oct 28;15(10):e104914. doi: 10.1136/bmjopen-2025-104914 (PMC12570927; doi:10.1136/bmjopen-2025-104914)
Supplement: online supplemental table 1 [file bmjopen-15-10-s001.docx]

**Supplementary table S1 Comorbidities below 5% of prevalence**

| **Comorbidities** | **Male** | **Female** | **Method** | ***P*- value** |
| --- | --- | --- | --- | --- |
| Malignancy | 62(1.2%) | 14(1.0%) | Chi squared | 0.443 |
| Left ventricular failure | 76 (1.5%) | 10 (0.7%) | Chi squared | <0.019 |
| Valvular heart disease | 97 (1.9%) | 12 (0.8%) | Chi squared | 0.005 |
| Lung cancer | 30 (0.6%) | 16 (1.1%) | Chi squared | 0.005 |
| Major depressive disorder | 33 (0.6%) | 26 (1.8%) | Chi squared | <0.001 |
| TB | 1 (0.0%) | 2 (0.1%) | Chi squared | 0.062 |
| COPD | 83 (1.6%) | 13 (0.9%) | Chi squared | 0.043 |

**Supplementary table S2 Independent Samples t-test Comparing predicted % of DLCO and FVC with or without hiatus hernia**

| **Variable** | **Levene’s Test (p)** | **t (df)** | **Mean Difference** | **95% CI** | ***P*-value** |
| --- | --- | --- | --- | --- | --- |
| **DLCO** | 0.139 | -1.631 (4581) | -1.578 | (-3.476, 0.319) | 0.103 |
| **FVC** | 0.916 | -2.179 (6521) | -1.940 | (-3.686, -0.195) | 0.029 |

**Supplementary table S3 Means Survival Time of 12-month duration of chest symptoms prior to baseline visit among females**

| Variable | Mean | | | |
| --- | --- | --- | --- | --- |
|  | Estimate years | Std. Error | 95% Confidence Interval | |
|  |  |  | Lower Bound | Upper Bound |
| Less than 12 months | 11.1 | 0.59 | 9.99 | 12.28 |
| More than 12 months | 8.1 | 0.26 | 7.60 | 8.60 |

Log rank (Mantel-Cox), chi-square 8.929, *p* value 0.003
