# Supplementary material for: Gender differences in clinical features, comorbidities and prognostic outcomes in idiopathic pulmonary fibrosis—a retrospective cohort analysis from the British Thoracic Society Interstitial Lung Disease Registry
Source: BMJ Open. 2025 Oct 28;15(10):e104914. doi: 10.1136/bmjopen-2025-104914 (PMC12570927; doi:10.1136/bmjopen-2025-104914)
Supplement: Supplementary file 2 [file bmjopen-2025-104914.reviewer_comments.pdf]

PEER REVIEW HISTORY

BMJ Open publishes all reviews undertaken for accepted manuscripts. Reviewers are asked to complete a checklist review form and are provided with free text boxes to elaborate on their assessment. These free text comments are reproduced below.

ARTICLE DETAILS

Title (Provisional)

Gender Differences in Clinical Features, Comorbidities, and Prognostic Outcomes in Idiopathic Pulmonary Fibrosis – A retrospective cohort analysis from the British Thoracic Society Interstitial Lung Disease Registry

Authors

Sia, Leng Cheng; Amanda, Gina; Bączek, Karol; Achaiah, Andrew; Sesé, Lucile; Chaudhuri, Nazia

---

VERSION 1 - REVIEW

**Reviewer**	**1**
**Name**	**Fu, Pin-Kuei**
**Affiliation**	**Taichung Veterans General Hospital**
**Date**	**31-May-2025**
**COI**	**None**

This study represents a large-scale retrospective cohort analysis based on the UK-wide British Thoracic Society (BTS) IPF/ILD Registry (2013–2024). The investigators included 6,666 patients with multidisciplinary team–confirmed idiopathic pulmonary fibrosis (IPF) diagnoses, across 91 hospitals and 74 regional healthcare trusts. The primary objective—to examine sex-based differences in the clinical presentation, comorbidities, and survival outcomes in IPF—addresses a timely and clinically relevant topic that may support the development of sex-specific, personalized management strategies.

Summary and Interpretation

The study design is straightforward and robust, utilizing a national registry with substantial representation. The results align with current literature: Males comprised 78% of the cohort and had higher rates of smoking, comorbidities (diabetes, ischemic heart disease), lower baseline FVC, and a higher rate of antifibrotic treatment use. Females were more likely to be free of comorbidities and had a higher prevalence of hiatus hernia (HH). Prognostic analysis revealed: In males: Poorer survival was associated with older age, IHD, lower FVC, and DLCO, while HH was a protective factor; In females: Symptom duration >12 months, oxygen therapy at baseline, and lower DLCO were poor prognostic markers, while GERD appeared

protective. Median survival was significantly longer in females (11.6 years vs. 8.3 years, $p < 0.001$).

Major Concerns and Recommendations

1. Lack of a Stated Hypothesis on Gender Inequity in IPF

Given the study's focus on sex-based clinical and prognostic differences, the authors should clearly articulate an underlying hypothesis addressing potential sex-based disparities in IPF diagnosis, treatment access, and outcomes. A more focused review of unmet needs in sex-specific IPF care—especially in the introduction or background—would strengthen the rationale for the study.

2. Impact of the UK Healthcare System on Observed Gender Differences

Many findings—such as the higher comorbidity burden and greater use of antifibrotic treatment in males—are consistent with prior literature. However, the role of the NHS prescribing framework, particularly historical criteria tied to lung function thresholds, may have influenced these trends. A discussion on how systemic healthcare factors may have contributed to observed sex differences would be valuable.

3. Survival Paradox: Higher Treatment Rate in Males Yet Worse Outcomes

Despite a higher rate of antifibrotic treatment in males, the median survival was 3.3 years shorter than in females. This paradox requires discussion. Could earlier diagnosis in females, biological differences in disease progression, or differences in response to therapy explain this disparity?

4. Symptom Duration >12 Months as a Poor Prognostic Factor in Females

The finding that longer symptom duration (>12 months) is a poor prognostic factor in females is noteworthy. The authors should report the proportion of female patients meeting this criterion and the median survival in this subgroup, which would help contextualize its clinical impact.

5. GERD and HH as Protective Factors: A Contradiction to Existing Literature

One of the most surprising findings is that GERD and HH were associated with improved survival—GERD in females, HH in males. These findings contrast with established hypotheses suggesting that chronic reflux and HH contribute to microaspiration and fibrosis progression. The authors must clarify:

o How were GERD and HH defined—by patient self-report, imaging (CT), endoscopy, or clinical diagnosis?

o Were these diagnoses standardized across centers?

o Could there be misclassification or selection bias, particularly given the low reported prevalence of HH (6.8%) compared to published rates of 40–50%?

**Reviewer**	**2**
**Name**	**Ozmen, Ipek**
**Affiliation**	**SaÄ□lÄ±k Bilimleri Ã□niversitesi SÃ¼reyyapaÅ□a gÃ¶Ä□Ã¼s hastalÄ±klarÄ± hastanesi, chest disease**
**Date**	**29-Jul-2025**
**COI**	**None**

The study aims to idetify gender differences in IPF patients. 6666 is a good number and study period is 11 years. Vast majority of the patients are male patients , lower female patients.

The authers mentioned prognostic factors between males and females, suggesting the need for

gender-specific approaches in the management of IPF. What would be these approches ?

Did you notive any socioeconomical or eduactional factor effecting patients' perception for IPF

VERSION 1 - AUTHOR RESPONSE

Reviewer: 1

Dr. Pin-Kuei Fu, Taichung Veterans General Hospital

Comments to the Author:

This study represents a large-scale retrospective cohort analysis based on the UK-wide British Thoracic Society (BTS) IPF/ILD Registry (2013–2024). The investigators included 6,666 patients with multidisciplinary team–confirmed idiopathic pulmonary fibrosis (IPF) diagnoses, across 91 hospitals and 74 regional healthcare trusts. The primary objective—to examine sex-based differences in the clinical presentation, comorbidities, and survival outcomes in IPF—addresses a timely and clinically relevant topic that may support the development of sex-specific, personalized management strategies.

Summary and Interpretation

The study design is straightforward and robust, utilizing a national registry with substantial representation. The results align with current literature: Males comprised 78% of the cohort and had higher rates of smoking, comorbidities (diabetes, ischemic heart disease), lower baseline FVC, and a

higher rate of antifibrotic treatment use. Females were more likely to be free of comorbidities and had a higher prevalence of hiatus hernia (HH). Prognostic analysis revealed: In males: Poorer survival was associated with older age, IHD, lower FVC, and DLCO, while HH was a protective factor; In females: Symptom duration >12 months, oxygen therapy at baseline, and lower DLCO were poor prognostic markers, while GERD appeared protective. Median survival was significantly longer in females (11.6 years vs. 8.3 years, $p < 0.001$).

Major Concerns and Recommendations

1. Lack of a Stated Hypothesis on Gender Inequity in IPF

Given the study's focus on sex-based clinical and prognostic differences, the authors should clearly articulate an underlying hypothesis addressing potential sex-based disparities in IPF diagnosis, treatment access, and outcomes. A more focused review of unmet needs in sex-specific IPF care—especially in the introduction or background—would strengthen the rationale for the study.

Original last paragraph of introduction:

Mortality rates in IPF are consistently higher in males than in females across multiple national cohorts.(1–4) Males with IPF also have worse transplant-free survival than females, with approximately a difference of 13 months in median transplant-free survival between genders.(3) The reasons underlying these gender differences are likely complex, including genetic, hormonal, and socioeconomic factors. These differences between genders in diagnosis and outcomes thus provides the rationale for our study. We aimed to investigate whether there are gender differences in the clinical presentation, comorbidities, and outcomes of IPF patients in a UK population.

Revised:

Mortality rates in IPF are consistently higher in males than in females across multiple national cohorts.(1–4) Males with IPF also have worse transplant-free survival than females, with approximately a difference of 13 months in median transplant-free survival between genders.(3) The reasons underlying these differences are likely multifactorial, including genetic, hormonal, behavioral, environmental, and socioeconomic influences. Potential inequities in presentation, diagnosis, treatment access, and clinical trial representation may further contribute to gender

disparities in outcomes. Evidence regarding sex differences in comorbidities and their impact on survival remains limited. We hypothesized that gender differences would be evident in baseline characteristics, comorbidities, and prognostic outcomes in a UK IPF population. By addressing this knowledge gap, our study aims to inform the development of more equitable, gender-sensitive management strategies.

2. Impact of the UK Healthcare System on Observed Gender Differences

Many findings—such as the higher comorbidity burden and greater use of antifibrotic treatment in males—are consistent with prior literature. However, the role of the NHS prescribing framework, particularly historical criteria tied to lung function thresholds, may have influenced these trends. A discussion on how systemic healthcare factors may have contributed to observed sex differences would be valuable.

Original 5th paragraph of discussion:

In our cohort, males were more frequently treated with antifibrotic treatment than females. FVC was lower in males, with values typically <78% but >80% in females. Similarly, studies from the U.S also report fewer females received antifibrotic treatment.(5, 6) There was no difference in duration of symptoms and HRCT finding between both genders, however the prescription patterns differed between genders. It is important to note that antifibrotic medications were restricted to patients with FVC <80% from 2013 to 2018. (7–9) Given that our cohort includes patients enrolled since 2013, this historical prescribing trend may have influenced treatment patterns. This is further supported by a Nordic study, which found that patients with higher FVC percentages are less likely to be prescribed antifibrotic drugs.(10)

Revised:

In our cohort, males were more frequently treated with antifibrotic treatment than females. FVC was lower in males, with values typically <78% but >80% in females. Similarly, studies from the U.S also report fewer females received antifibrotic treatment.(36, 37) Despite similar duration of symptoms and HRCT findings between genders, indicating no diagnostic delay or imaging-based bias, prescription patterns differed. Health-seeking behaviour is therefore unlikely to explain the disparity.

Instead, antifibrotic prescribing in the UK was historically restricted to patients with an FVC <80% between 2013 and 2023, under National Institute for Health and Care Excellence guideline. Nintedanib for patients with FVC >80% was initially deemed not cost-effective, as the incremental cost-effectiveness ratios exceeded the thresholds considered acceptable for National Health Service resources. Given that our cohort includes patients enrolled since 2013, these historical prescribing restrictions likely influenced treatment patterns and may have contributed to the observed gender differences. (38–40)

3. Survival Paradox: Higher Treatment Rate in Males Yet Worse Outcomes

Despite a higher rate of antifibrotic treatment in males, the median survival was 3.3 years shorter than in females. This paradox requires discussion. Could earlier diagnosis in females, biological differences in disease progression, or differences in response to therapy explain this disparity?

Original: (not included)

Revised:

The survival paradox was evident in our cohort, as males had a median survival 3.3 years shorter than females despite being more frequently prescribed antifibrotic therapy. This real-world finding highlights that higher prescription rates do not necessarily translate into improved survival outcomes. Disease progression in IPF is not solely determined by antifibrotic use; other confounding factors play a crucial role. In our cohort, males presented with a higher comorbidity burden, lower baseline FVC, and a greater history of smoking, all of which are associated with poorer prognosis

4. Symptom Duration >12 Months as a Poor Prognostic Factor in Females

The finding that longer symptom duration (>12 months) is a poor prognostic factor in females is noteworthy. The authors should report the proportion of female patients meeting this criterion and the median survival in this subgroup, which would help contextualize its clinical impact.

Original: not mentioned.

Revised:

Approximately two-thirds of patients presented with symptoms for more than 12 months prior to the baseline visit, with no significant differences between sexes. Among females, longer symptom duration was associated with a mean survival that was 3 years shorter compared with those who presented earlier (Supplemental Table S3). Prolonged symptom duration may reflect delayed initiation of antifibrotic therapy.

Hoyer N, et al. reported that in patients with baseline FVC >80% predicted, longer diagnostic delay has a greater impact on progression-free survival after diagnosis.(11) Our study supports this observation, as symptom duration appeared to have a stronger impact among females with higher baseline FVC in our cohort.

New reference: Hoyer N, Prior TS, Bendstrup E, Shaker SB. Diagnostic delay in IPF impacts progression-free survival, quality of life and hospitalisation rates. BMJ Open Respir Res. 2022; 9(1):1–8.

5. GERD and HH as Protective Factors: A Contradiction to Existing Literature

One of the most surprising findings is that GERD and HH were associated with improved survival—GERD in females, HH in males. These findings contrast with established hypotheses suggesting that chronic reflux and HH contribute to microaspiration and fibrosis progression. The authors must clarify:

o How were GERD and HH defined—by patient self-report, imaging (CT), endoscopy, or clinical diagnosis? GERD and HH were captured through patient self-reported clinical diagnosis and clinician data entry within a prospective registry. Since 2013, the standardized proforma used across all centers has included specific fields for “known hiatus hernia” and “symptoms of GERD.

o Were these diagnoses standardized across centers?

Yes. All participating centers used the same standardized proforma, ensuring consistency in data collection, however specific definitions of these diagnosis were not provided to clinicians and assessment was left to clinicians assessment which could introduce variations in practise.

o Could there be misclassification or selection bias, particularly given the low reported prevalence of HH (6.8%) compared to published rates of 40–50%?

We re-checked the raw data and confirmed that the reported prevalence of HH (6.8%) was genuine. The lower prevalence compared to published rates likely reflects methodological differences. Published rates of 40–50% are typically based on CT imaging among IPF patients, whereas in our registry HH was identified by patient self-reported clinical diagnosis and clinician entry. Thus, under-reporting is possible in our cohort..

Revised from original discussion section:

In contrast to few studies reporting a high prevalence of HH, up to 42-53% in IPF patients, our cohort demonstrated a significantly lower incidence of HH at 6.8%, with a higher prevalence observed in females.(28, 42, 43) HH was captured prospectively by patient self-reported clinical diagnosis and clinician entry in a standardized proforma used across all centers since 2013, whereas published prevalence rates are typically derived from CT. As such, under-reporting is possible in our cohort. C. Tossier et al and J.A. Mackintosh et al highlight an association between HH and poor survival in IPF. However, our data suggests that the presence of HH in males may be a protective factor. Some studies found no clear link between HH and mortality, which might indicate that HH does not significantly impact survival outcomes in certain IPF cohorts.(42, 44) HH is a condition caused by translocation of stomach above diaphragm as well as widening of esophageal hiatus which could be partly caused by degeneration over time affecting the compositional elastin and collagen fibers or due to pressure gradient in between intrathoracic and intra-abdominal.(47) Interestingly, the presence of HH was significantly associated with higher baseline FVC in our cohort. (supplemental table S2) ~~The reason of how HH is a protective factor in males requires further exploration~~. These conflicting data ~~could be attributed to differences in patient population, or the study methodologies employed and~~ requires further exploration.

GERD closely related to HH has been shown to be associated with improved survival.(42, 45, 46) This is consistent with our findings, where GERD appeared to be associated with improved survival in females. Whether proton pump inhibitor (PPI) treatment contributes to this effect remains uncertain and has been a topic of controversy for decades.

Reviewer: 2

Dr. Ipek Ozmen, SaÄŸlÄ±k Bilimleri Ãœniversitesi SÃ¼reyyapaÅŸa gÃ¶ÄŸÃ¼s hastalÄ±klarÄ± hastanesi

Comments to the Author:

The study aims to idetify gender differences in IPF patients. 6666 is a good number and study period is 11 years. Vast majority of the patients are male patients , lower female patients.

The authers mentioned prognostic factors between males and females, suggesting the need for gender-specific approaches in the management of IPF. What would be these approches ?

Original conclusion:

In conclusion, our findings emphasize the importance of a gender-specific approach to the diagnosis and management of IPF. Distinct prognostic indicators between males and females suggest that clinical decisions may benefit from tailoring to gender-related disease patterns. Future prospective studies are warranted to further explore these differences and evaluate their implications for IPF management.

Revised conclusion:

In conclusion, our findings emphasise the importance of a gender-specific approach to the diagnosis and management of IPF in view of distinct prognostic indicators between males and females. For example, earlier screening and referral pathways may be warranted in women presenting with unexplained dyspnoea or cough, whereas a male-specific focus could prioritise smoking cessation programmes and optimisation of cardiovascular risk. Future prospective studies are warranted to further explore these differences and evaluate their implications for IPF management.

Did you notive any socioeconomical or eduactional factor effecting patients' perception for IPF

Response:

Thank you for your comment, the BTS registry does not capture patients' perception for IPF.

VERSION 2 - REVIEW

**Reviewer**	**1**
**Name**	**Fu, Pin-Kuei**
**Affiliation**	**Taichung Veterans General Hospital**
**Date**	**05-Oct-2025**
**COI**	

The authors have thoroughly addressed all of my comments and made substantial revisions in this version of the manuscript, resulting in a more coherent structure and improved readability. Therefore, I agree that this version of the manuscript should be accepted for publication.
